# Heatwaves exacerbate pollen limitation through reductions in pollen production and pollen vigour

**DOI:** 10.1093/aobpla/plae045

**Published:** 2024-09-12

**Authors:** Nick M Rosenberger, Jeremy A Hemberger, Neal M Williams

**Affiliations:** Graduate Group in Ecology, University of California – Davis, 1 Shields Ave, Davis, CA 95616, USA; Department of Entomology and Nematology, University of California – Davis, 1 Shields Ave, Davis, CA 95616, USA; Department of Entomology and Nematology, University of California – Davis, 1 Shields Ave, Davis, CA 95616, USA; Department of Entomology, University of Wisconsin – Madison, 1630 Linden Dr, Madison, WI 53706, USA; Department of Entomology and Nematology, University of California – Davis, 1 Shields Ave, Davis, CA 95616, USA

**Keywords:** *Brassica napus*, climate change, heat, heatwaves, pollen limitation, pollen quality, pollination

## Abstract

Increasingly frequent heat waves threaten the reproduction of flowering plants; compromising the future persistence, adaptive capacity, and dispersal of wild plant populations, and also the yield of fruit-bearing crop plants. Heat damages the development of sensitive floral organs and gametes, which inhibits pollen germination, pollen tube growth, and fertilization. However, the role of heat has not been integrated into the framework of pollen quantity and quality limitation and how heat influences the success of cross and self-pollination. We exposed developing flowers to either controlled temperature (25 °C:20 °C) or extreme heat (35 °C:20 °C) over 72 h. We then hand-pollinated them with either crossed or self-derived pollen from the same temperature treatment to determine the direct and interactive effects of simulated heatwaves on pollen tube growth and resulting seed set. We also collected anthers from virgin flowers to measure heat impacts on pollen production. Under cooler control temperatures pollen tube survival of self-derived pollen was approximately 27% lower than that of crossed pollen. Pollen tube survival in heat-treated cross-pollinated and heat-treated self-pollinated flowers were 71% and 77% lower compared to flowers cross-pollinated at control temperatures. These differences in pollen tube survival rate *between* heat-treated cross-pollinated and heat-treated self-pollinated flowers were insignificant. Furthermore, extreme heat reduced seed set by 87%, regardless of pollen origin, and also reduced pollen production during flower development by approximately 20%. Our results suggest flowers that develop during heatwaves are likely to experience exacerbated pollen quantity and quality limitation driven by changes in pollen production and pollen vigour. Heatwave-induced pollen limitation will likely reduce crop yields in agricultural systems, and depress mating and reproduction in wild plant species, the latter of which may hinder the adaptive capacity of plants to a rapidly changing world.

## Teaser

Flowering plants depend on successful pollen dispersal within and among flowers to produce seeds, but this process may be highly vulnerable to the effects of climate change and more frequent heatwaves due to flower sensitivity to high temperatures. Heat may ultimately affect the dynamics of pollination when heat coincides with flower development and the formation of gametes (pollen and ovules) and reproductive organs, which affects both the quantity of gametes available, and their vigour and viability. We found that heat during floral development primes flowers to experience shortfalls in pollination by reducing pollen production and pollen vigour. These effects from heat also reduced the survival rate of pollen tubes from deposited pollen grains which subsequently diminished seed set in flowers exposed to heat. Our work suggests that as climate change intensifies, plants that flower concurrently with heatwaves will have greatly diminished mating opportunities and reproductive success.

## Introduction

Heatwaves are becoming more frequent, intense, and prolonged as climate warming increases global average temperatures and drives climate instability (Meehl and Talbaldi 2004; Stillman 2019; Thiery *et al.* 2021). These amplified heatwaves expose organisms to high temperatures that may disrupt sensitive life history events, as well as alter their interactions with other organisms (Jamieson *et al.* 2012; Pincebourde and Casas 2019) by pushing them beyond their thermal optima (Harvey *et al.* 2023). Sexual reproduction of angiosperms is one life history event that is highly sensitive to extreme heat (Hedhly *et al*. 2009). When heatwaves coincide with flowering, high temperatures can disrupt floral development, alter the dynamics of pollination (Nicholson and Egan 2020) and reduce plant reproduction (Hedhly *et al.* 2009; Hedhly 2011), which have important implications for plant demography (Jiménez *et al.* 2011), and global food production (Battisti and Naylor 2009).

Extreme heat during flower development can damage floral reproductive organs (both stamens and pistils; Young *et al.* 2004; Hedhly *et al.* 2009), reducing overall male and female functioning at multiple stages of the pollination and fertilization processes. First, heat during development can disrupt gametogenesis such that flowers produce fewer ovules and pollen grains (Young *et al.* 2004; Hedhly *et al.* 2005, 2009; Koti *et al.* 2005; Distefano *et al.* 2018). This limits the number of pollen grains that can be dispersed to recipient flowers and the number of ovules they can fertilize. Second, heat during flower development can reduce pollen viability and vigour; reducing pollen germination and pollen tube growth (Young *et al.* 2004; Koti *et al.* 2005; Hedhly *et al.* 2009; Shi *et al.* 2018). Third, heat can compromise stigma receptivity and stylar tissue in some flowering plant species (Prasad *et al.* 2002; Hedhly *et al.* 2005, 2009; Hedhly 2011; Distefano *et al.* 2018), which affects the germination of pollen grains on stigmas and the maternal flower’s capacity to support pollen tube growth to the ovules (Young *et al.* 2004; Hedhly *et al.* 2009; Zinn *et al.* 2010). Previous studies using reciprocal crossing found that although heat effects on pollen, stigmas, and styles all impacted reproductive outcome, the effect of heat on pollen more strongly reduced reproductive success (Monterosso and Wein 1990; Peet *et al.* 2002; Young *et al.* 2004). Thus, although previous studies (predominantly using crop plants) have identified underpinning mechanisms by which extreme heat diminishes reproduction, such responses have not been integrated into the framework of floral ecology, and how heat’s effects on pollen availability, pollen vigour and pollen tube survival may exacerbate pollen limitation.

Pollen limitation occurs when seed production is limited by pollen receipt instead of resource availability (Ashman *et al.* 2004; Knight *et al.* 2005). This limitation develops via two general pathways, referred to as quantitative pollen limitation and qualitative pollen limitation (Aizen and Harder 2007; Harder and Aizen 2010). Quantitative pollen limitation occurs when an insufficient number of pollen grains are received on stigmas to fertilize the available ovules that the plant has and can otherwise mature into seeds (Ashman *et al.* 2004; Knight *et al.* 2005; Aizen and Harder 2007). In contrast and under normal environmental conditions, qualitative pollen limitation arises when low-quality pollen is deposited on stigmas and limits the successful fertilization and production of seeds. Pollen quality is a function of the pollen itself and the health of the siring plant (Aizen and Raffaele 1998), but also relative to the maternal plant on which it is deposited (Souto, Aizen and Premoli 2002; Aizen and Harder 2007; Harder *et al.* 2016). As such, low pollen quality can result from a failure of pollen to germinate either because of low pollen viability or stigma–pollen incompatibility, as well as from depressed pollen tube survival, post-fertilization incompatibility, or strong inbreeding depression (Jóhannsson *et al.* 1998; Aizen and Harder 2007; Harder *et al.* 2016). Generally, self-pollination represents the extreme of pollen donor and maternal relatedness and is subject to a range of self-incompatibility filtering processes that operate at germination, pollen tube growth, fertilization, or post-zygotic stages (Kao and McCubbin 1996; Aizen and Harder 2007; Harder *et al.* 2016). Consequentially, flowers usually need to receive more self-pollen grains per ovule relative to cross pollen grains per ovule for complete ovule fertilization within self-compatible plant species or plants with mixed mating systems (Aizen and Harder 2007; although see Cruzan and Barett 2016 for cryptic self-incompatibility).

**Figure 1.**
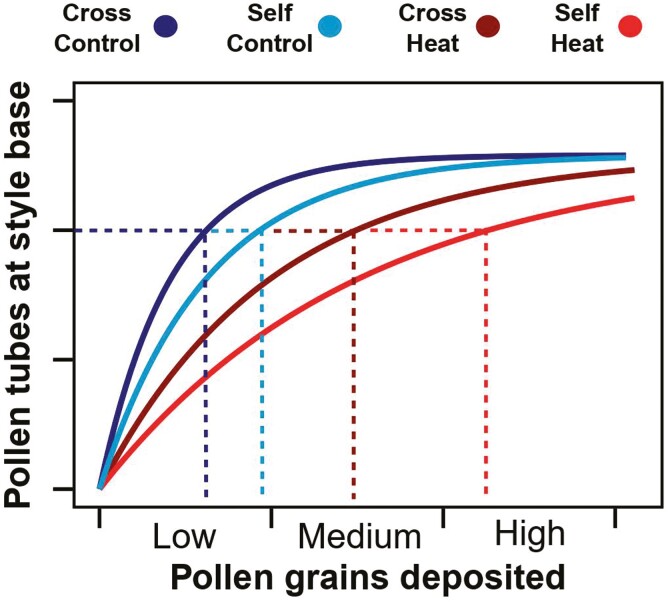
The graphic represents the relationship between the number of pollen grains deposited and the number of pollen tubes surviving to the base of the style which may be able to fertilize ovules. Colour represents the conditions of pollen during pollen development. Dashed lines indicate predictions of required pollen receipt to produce the same number of pollen tubes to reach the base of the style based on heat’s effects on pollen quality through pollen origin and temperature treatment. Bins on the × axis indicate regions qualitatively considered low, medium, and high pollen receipt. A color version of this figure appears in the online version of this article.

Regardless of pollen origin, pollen tubes that germinate compete for limited space within stylar tissues. Competition intensifies as higher numbers of pollen grains are deposited (Cruzan 1986, 1989; Harder, Aizen, and Richards 2016; Harder, Aizen, Richards, *et al.* 2016), creating a non-linear, negative-exponential relationship between pollen deposition and final pollen tube survival (Fig. 1: derived from Aizen and Harder 2007; Harder *et al.* 2016). This relationship is an essential feature of the post-pollination process, as it dictates how many pollen tubes can fertilize ovules and to some extent become seeds. Saturation is reached with fewer, higher quality pollen grains than with lower quality pollen grains (here presented as cross and self; Fig. 1). The saturation curve also illustrates how pollen quantity limitation constrains pollen tube survival at low levels of pollen deposition, regardless of pollen origin, whereas pollen quality limitation via pollen origin constrains pollen tube survival at high levels of pollen deposition.

Although both pollen quantity and quality limitations exist under normal environmental conditions, the negative effects of extreme heat on pollen production, viability, and vigour, as well as on stylar tissues may exacerbate the likelihood of a plant experiencing pollen limitation (Young *et al.* 2004). When heat reduces pollen production it can increase the risk of pollen quantity limitation by diminishing the pool of pollen that is available for dispersal among receptive flowers and reducing the average quantity of pollen deposited onto stigmas (Cresswell 1999). In contrast, heat-induced decreases in pollen viability and vigour, and stylar support of pollen tube growth should further limit pollen tube survival beyond pre-existing differences in quality that emerge from pollen origin (cross versus self: Fig. 1). We assume that heat-induced decreases in pollen vigour and stylar support of pollen tube growth have additive negative effects on the risk of pollen limitation. Therefore, to maximize pollen tube survival, heat-stressed flowers would need to receive proportionately more cross-pollen grains, and even more self-pollen (red lines—Fig. 1).

Considering extreme heat’s effects on flowers, it is likely that flowers which develop during heatwaves will be at greater risk of pollen limitation. Importantly, such conditions in the mating environment may also be exacerbated by the negative effects heat can have on pollinators and their ability to pollinate flowers (Walters *et al.* 2022; Hemberger *et al.* 2023). Illuminating how heat influences the dynamics of quantitative and qualitative pollen limitation from the perspective of plants is critical to understanding how both wild plant populations and fruit-bearing crops will be affected by ongoing climate change and heatwave events. To address this, we simulated heatwave conditions using chamber experiments and hand pollination to ascertain how extreme heat affects pollen quality and quantity limitation and reproductive success in *Brassica napus*. *Brassica napus* is an optimal species to study because it is an annual plant with a mixed mating system (self-compatible and produces seeds from self and cross-pollination; Okamoto *et al.* 2007). Thus, it allows us to parse heat’s effects on pollen limitation and its potentially additive effects on pollen origin (quality). From our framework, we predict that:

1. Extreme heat will impose qualitative pollen limitation on flowers via a reduction in pollen viability and vigour, increasing the number of pollen grains needed to overcome pollen limitation.
2. The effect of extreme heat on pollen viability and vigour will be additive to differences in pollen tube survival that already exist between cross- and self-pollen.
3. Extreme heat will decrease the number of pollen grains produced per flower, increasing the risk of quantitative pollen limitation through a reduction in pollen production.

## Methods

### Experimental heat treatments

**Figure 2.**
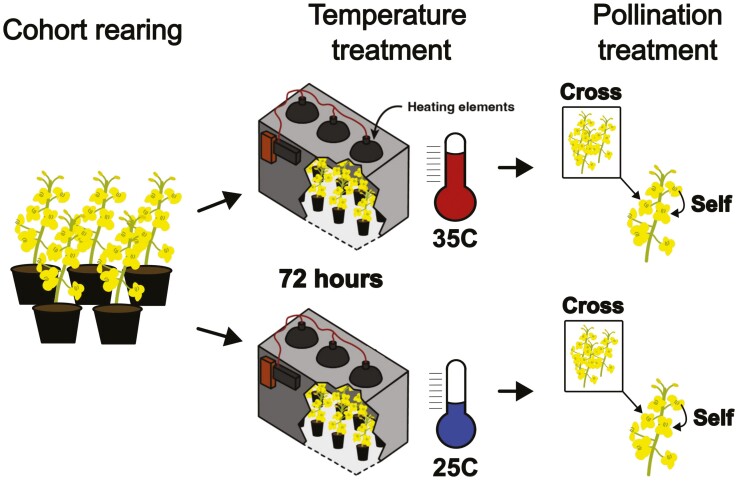
Figure presents the experimental process used during the study, which involved the plant rearing stage, temperature treatment phase and pollination phase. Plants we reared in cohorts within a greenhouse and then after flowers began to open were moved into the heating chamber (35 °C) or control chamber (25 °C) for temperature treatment. After temperature treatment of 72 h, plants were moved back into the greenhouse and were hand pollinated the following morning with cross and self-pollination.

We exposed *B. napus* plants to experimental heatwaves in growth chambers. We collected anthers to measure pollen production and performed hand pollination using cross and self-pollen on plants from the same experimental temperature treatments to assess extreme heats’ effect on pollen quality and quantity limitation (Fig. 2). We used seeds of rapid cycling *B. napus* sourced from the Rapid Cycling Brassica Collection University of Wisconsin (Stock #W5–1), which are not inbred lines and retain considerable genetic diversity (P. H. Williams, personal communication).

We seeded cohorts of plants in Vigoro All-Purpose Potting Mix (Vigoro, Lake Forest, Illinois, USA) in 4-inch pots and initially grew them inside under full spectrum fluorescent lights between February and March 2021 at the University of California-Davis, California, USA. Once their first true leaves had developed we moved plants to a greenhouse where they were kept at 25 °C on a 16/8 h day/night cycle, watered daily, and fertilized every 3 days with Peters Professional Fertilizer with micronutrients at 1:1:1—N:P:K at 100 ppm volume. When plants began to develop branching racemes (approximately at universal growth stage 61–63 see Lancashire *et al.* 1991), they were moved into treatment chambers for experimental heating during April and May 2021.

From each of 20 plant cohorts we placed nine plants in an experimental heating chamber and nine plants into a replica control chamber for the temperature treatment trial (Figure 2). Plants were kept in their respective treatment chambers for 72 h and under the same day-night cycle with full-spectrum fluorescent lights. In the heating chamber, ceramic heat lamps were used to elevate daytime temperatures to ~35 °C to simulate heatwave conditions. During the night cycle, the heating elements were turned off. We chose this temperature because it is at the edge of thermal tolerance for *B. napus* (Young *et al.* 2004; Lohani *et al.* 2022), and represents a moderate heatwave in its growing regions. After the treatment period, we collected the anthers of one flower from each plant to measure pollen production (see below).

### Hand pollination experiments

We conducted hand pollination experiments the morning after temperature treatments (~12 h after) on emasculated virgin flowers to measure pollen limitation and effects of heat on cross and self-pollen success. Flowers chosen had recently opened and their anthers had not yet dehisced. Emasculation of anthers also precluded the possibility of self-pollen grains being deposited on the stigmas of cross-pollinated flowers, and any autogamous pollination. We purposefully varied the amount of pollen transferred across replicates to capture the asymptotic nature of the deposition-pollen tube growth relation (Fig. 1). To quantify the impacts of pollen origin on pollen quality within the context of heatwaves, plants that had either been exposed to heat or normal temperatures were hand pollinated using either cross pollen or self-pollen. Cross pollen was always from donors in the same trial so that heat-stressed recipient plants were pollinated by heat-stressed donor plants and control plant recipients were pollinated by control plant donors. When possible, we pollinated two virgin flowers on each plant: one was cross-pollinated and another was self-pollinated so that differences among plants could be accounted for. To cross-pollinate, we put anthers from three plants (one flower per plant) into an Eppendorf tube and shook it to mix the pollen. We then used a cotton swab to pick up pollen and deposit it on the stigma of the virgin flower. We repeated this with a fresh Eppendorf tube for self-pollination but used anthers from three self-originating flowers. We placed varying amounts of pollen on each stigma to create a range of pollen deposition values from our data set to ensure capturing the saturating negative exponential function we hypothesized exists in Fig. 1. We visually inspected stigmas during hand pollination to monitor our efforts for variation in pollen deposition. After hand pollination was complete, the flowers were left for 24 h to ensure pollen tube growth was complete (Young *et al*. 2004). We then collected styles and stored them in 1.5 mL Eppendorf tubes filled with 70% Ethanol.

### Pollen deposition and pollen tubes

To prepare style samples for staining, we rinsed them with DI water and then left them to soak for 1 h before being transferred to 8M NaOH solution. Once transferred into the NaOH solution, we heated styles on a hot plate for 1 h at 55 °C to soften stylar tissues. We then rinsed styles again with DI water and soaked them for 1 more hour before moving them to a buffer of K_3_PO_4_ 0.1M with 1% aniline blue dye for staining (mixed at 90% K_3_PO_4_ and 10%, 1% aniline blue). We then kept styles in the refrigerator for 24 h to stain thoroughly before squashing them on microscope slides for observation under a fluorescent microscope (Martin 1959). Under fluorescent light, we counted the number of pollen tubes at the base of the style, and the number of pollen grains deposited on the stigma. Although some pollen tubes may not have fluoresced, this should not be biased between treatments. These measurements together inform at the individual flower level how much pollen is required to overcome the effects of heat and pollen origin on pollen quality limitation.

### Pollen production

After temperature treatments, we collected all six anthers from individual flowers of various ages, with each flower selected haphazardly from each plant to assess the impact of heat on pollen production. We placed collected anthers from individual flowers in 70% ethanol inside 1.5 mL Eppendorf tubes for later processing and counting. Importantly, we acknowledge that flowers that opened at the onset of the simulated heatwave would have already developed pollen grains, so we may underestimate the full effect of heat on pollen production. However, we also recognize that this method more accurately represents the pollen available for dispersal on a plant after 72 h of heat exposure.

We used a Coulter Multisizer 3 Particle Counter (Beckman Coulter, Brea, CA, USA) to count pollen grains from anthers that had been stored in 70% ethanol. First, we vortexed Eppendorf tubes containing anthers, transferred their contents into a coulter cuvette, and sonicated them using a Microson^TM^ Ultrasonic Cell Disruptor (Misonix, Farmingdale, NY, USA) to release any residual pollen grains within anthers. We then rinsed individual anthers into the cuvette with 0.9% saline solution. For each cuvette, we weighed the empty weight of the cuvette and the final weight of the cuvette to calculate the sample volume. We counted the number of particles between 20 and 30 um within three, 1 mL samples of solution. We averaged the number of pollen grains from the three samples and multiplied it by the weight of the sample to calculate the total pollen grains per flower.

### Seed collection

We collected fruits from hand pollination experiments at fruit maturity when siliques were beginning to yellow and placed them in small coin envelopes. After the fruits had completely dried, we counted the seeds from each fruit. We collected all fruits that had been hand-pollinated in the experiment and counted the number of developed seeds in each fruit to measure the seed set.

### Data analysis

We conducted all analyses in R (v4.3.1). We used the package nlme (Pinhiero *et al.* 2023) to fit nonlinear mixed models to assess the relationship between pollen deposition and pollen tubes reaching the base of the style, building on a previously documented functional form (Aizen and Harder 2007). We used the package glmmTMB (Brooks *et al.* 2017) to analyse pollen production and seed set as general linear models, and generalized linear mixed models, respectively.

#### Pollen quality.

 Previous work using other species from different angiosperm families documented a consistent asymptotic relationship between pollen deposition on stigmas and pollen tubes reaching the base of styles (Aizen and Harder 2007; Harder, Aizen, and Richards 2016). Like in those previous explorations, we modelled pollen tube survival as a decelerating asymptotic function of pollen deposition (Equation 1) where *T* is the number of pollen tubes surviving to the base of the style, α is the asymptote, *r* is the rise of the function, and *P* is the number of pollen grains on the stigma.


$$\begin{array}{l} T\;=\;\alpha \;(1-e^{-r\;*\;P}) \end{array}$$

We fit this functional form to our data and compared the various parameters among our temperature and pollen origin treatments. We used likelihood ratio tests between three nested models to test our hypotheses about the impact of heat and pollen origin on pollen quality. The null model considered pollen tubes reaching the base of the style only as a function of pollen deposited on stigmas, the second a single-factor temperature treatment model (control and heat), and the third a two-factor temperature*pollen origin model. All three models included temperature treatment trials as a random effect rather than plant because plant observations consisted of only 1–2 flowers. This temperature*pollen origin model estimates an individual rise (*r*) parameter for each level of the temperature and pollen origin interaction. In our models, we chose to fix the asymptote (α) to a value estimated solely from cross-control plants and then set this limit (α=25.8). We estimated this by allowing the model to find independent rises and asymptotes for each treatment (see Appendix 1). This choice reflects a conceptual understanding of pollen deposition on stigmas and analytically allows for a clearer interpretation of heat and origin impacts (in the framework of Fig. 1). Conceptually, the choice suggests that, with sufficient deposition, the number of pollen tubes from lower quality sources reaching the base of the style will eventually converge on that from high-quality sources if sufficient pollen is available. We recognize that stigma clogging from low-quality pollen could disrupt this accumulation (Snow *et al.* 2000; Barrett 2002). Using a fixed asymptote assumes that the effect of heat is only influencing pollen viability and pollen tube growth and that it does not affect the maximum number of pollen tubes a style can support. Analytically, having an independent rise and asymptote in the model also would have required the maximum likelihood estimation to solve for both simultaneously. Because of the nature of this estimation, solving simultaneously may cause one parameter to overpower the other parameter when looking for the best fit and mask its effect. Therefore, we used a fixed asymptote so that we could clearly identify the effects of our treatments on pollen quality via the rise (*r*) parameter. For model fitting, we also constrained observations to those where there were 400 or less pollen grains deposited on the stigma. We constrained pollen deposition values to <400 because it encompassed the range where qualitative effects emerge and represented 75% of all observations. In the control treatments, pollen deposition ranged from 2 to 1225 grains and in the heat treatments 0–561 grains. Constraining the model also aided model convergence. We used pairwise comparisons based on the temperature * pollen origin model between *r* parameter estimates to assess statistical effects between temperature and pollen origin effects. Comparisons between the *r* estimates allowed us to address pollen quality explicitly under the assumption that the treatments shared similar limits to pollen tube survival.

#### Pollen quantity.

To test for differences in pollen production between heat and control plants, we used a general linear model assuming a normal distribution for the number of pollen grains per flower. We included temperature treatment as a fixed effect such that differences in pollen grains per flower indicate the effect of the temperature treatment (control vs. heat).


$$\begin{array}{l} pollen\;\;\;grains\;\;\;per\;\;\;f\;\;lower\;∼\;temperature \end{array}$$

Because each observation for this analysis came from an individual plant, we do not include a random effect as observations are assumedly independent.

#### Seed set.

To test differences in seed set from heat and control plants, and cross and self-pollination, we fit a generalized linear model assuming a negative binomial distribution, with an interaction between temperature treatment and pollen origin. We included a temperature treatment trial as a random effect for the same reason as stated in the pollen quality section.


$$\begin{array}{l} seed\;\;\;set\;∼\;temperature\;*\;pollen\;\;\;origin\;+\;(1|\;\;\;trial) \end{array}$$

From this model, we estimated marginal means using the emmeans package (Lenth *et al.* 2023), and used pairwise comparisons to compare differences in means between treatments. Differences between means in the pairwise comparisons indicate the relative effects of pollen origin (cross vs. self), and temperature treatment (heat vs. control) on seed production. We also examined model residuals and used dispersion tests using the DHARMa package to assess model fit.

## Results

**Figure 3.**
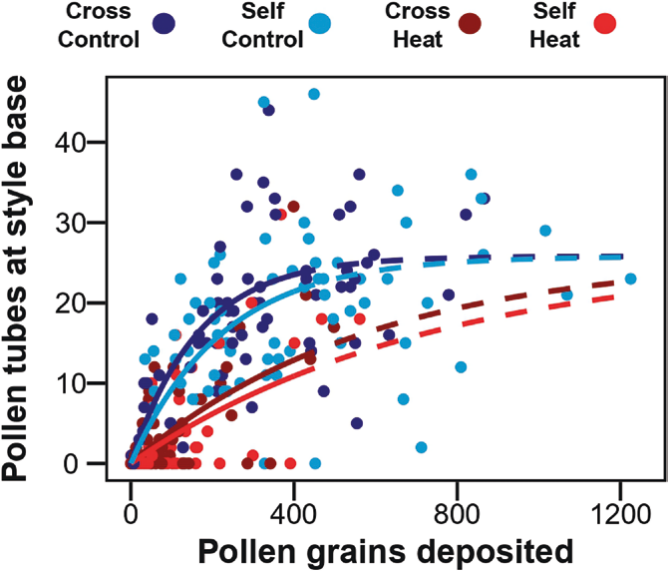
Model predicted values between pollen tubes reaching the base of the style and the number of pollen grains deposited. Trend lines are estimated from a model subset to <400 pollen grains which utilizes a common fixed asymptote estimated for control- and cross-pollinated plants in the full model. Colour represents pollination treatment and temperature treatment. Solid lines represent the relationship for the range of modelled data, whereas dashed lines represent extrapolation of the model not fit to data. A color version of this figure appears in the online version of this article.

Pollen origin and heat significantly affected pollen tube survival. Comparing among models of the relation between pollen deposition and pollen tubes, the model that included temperature treatment better explained variation than the null model (Table 1, *P* < 0.001). However, the model including temperature and pollen origin together was superior to the temperature only model (Table 1, *P* < 0.05) and the null model (Table 1, *P < *0.001). Pairwise comparisons of *r* (rise) among treatments from the temperature*pollen origin model showed that heat significantly decreased the rise of pollen tube survival relative to control plants and diminished the differences between cross and self-pollination (Tables 2 and 3, Fig. 3). The cross-pollinated control treatment had the steepest rise (*r*), whereas the self-pollinated control treatment rise was relatively 27% lower. When treated with heat, cross-pollinated flowers had a 71% lower rise, and self-pollinated flowers had a 77% lower rise relative to the cross-pollinated control.

**Table 1.** Results of pollen tube survival models and subsequent log-likelihood ratio tests. Null, temperature and full models represent the models tested in pollen tube survival analyses. Null model fits equation 1 without modifications of temperature treatment or pollination treatment. Temperature model includes only temperature treatment as a fixed effect. Full model includes the interaction between temperature and pollination treatment.

Model	df	AIC	Log- likelihood	Model comparison	Likelihood-ratio	*P* values
Null	3	1181.06	−587.53			
Temperature	4	1164.42	−578.21			
Full	6	1160.56	−574.28			
				Null vs. temperature	18.64	*P* < 0.001
				Null vs. full	26.49	*P* < 0.001
				Temperature vs. full	7.85	*P* < 0.05

**Table 2.** Pairwise comparisons of temperature * pollen origin treatments for *r—*pollen tube survival rate. In the pairwise comparison column, the level of the interaction indicated and which level it is being compared to.

Pairwise comparison	*T* ratio	*P* value
Heat * self vs. control * self	−3.72	*P* < 0.01
Heat * self vs. heat * cross	−0.88	*P* = 0.82
Heat * self vs. control * cross	−5.25	*P* < 0.01
Control * self vs. heat * cross	3.27	*P* < 0.01
Control * self vs. control * cross	−2.84	*P* < 0.05
Heat * cross vs. control * cross	−4.83	*P* < 0.01

*Note*: Number of surviving pollen tubes tests pairwise comparisons between model estimates of *r* (see Equation 1).

**Table 3.** Pairwise comparisons of marginal means of seed set for temperature * pollen origin treatments. In the pairwise comparison column, the level of the interaction indicated and which level it is being compared to.

Pairwise comparison	*T* ratio	*P* value
Heat * self vs. control * self	−6.84	*P* < 0.01
Heat * self vs. heat * cross	−0.76	*P* = 0.87
Heat * self vs. control * cross	−6.75	*P* < 0.01
Control * self vs. heat * cross	6.23	*P* < 0.01
Control * self vs. control * cross	0.14	*P = *0.99
Heat * cross vs. control * cross	−6.15	*P* < 0.01

**Figure 4.**
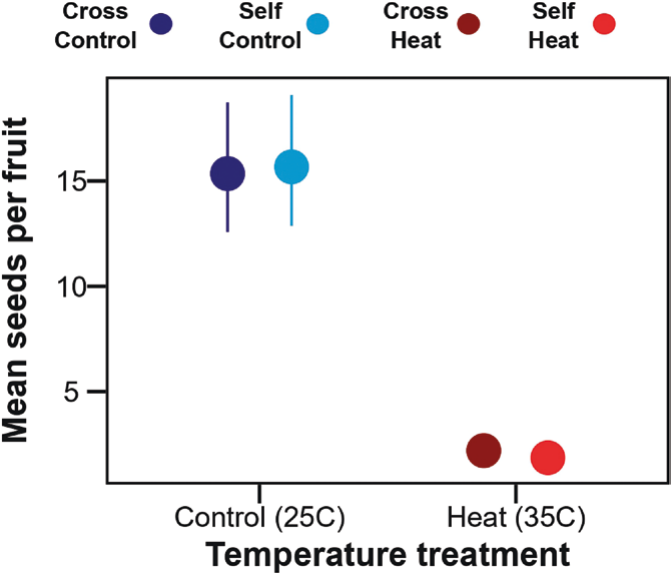
Estimated marginal mean number of seeds per fruit (± SE) from temperature and pollen origin treatments. Colour represents pollen origin treatment and temperature. A color version of this figure appears in the online version of this article.

Extreme heat also significantly reduced seed set (Figure 4, see Supporting Information—**Table S1**, *P* < 0.001,), and this effect did not depend on pollen origin (see Supporting Information—**Table S1**, temperature treatment × pollen origin interaction *P* = 0.45). In control-treated flowers, cross-pollination produced on average 15.35 ± 1.22SE seeds, whereas self-pollination produced 15.67 ± 1.22SE. In contrast in heat-treated flowers, cross-pollinated flowers produced on average 2.19 ± 1.28SE seeds, whereas self-pollination produced 1.85 ± 1.27SE seeds.

Heat reduced the amount of pollen produced per flower by 20% compared to those not exposed to heat stress (see Supporting Information—**Figure S1**, **Table S1**, *P* < 0.001). Control flowers produced on average 21 780.13 ± 1124.14SE pollen grains, whereas heat-treated flowers produced 17 329.19 ± 1146.18SE pollen grains.

## Discussion

Our results demonstrate that heat can strongly increase the risk of pollen limitation through its effects on pollen at multiple stages of the pollination process (pollen production, pollen tube survival, and seed set). As we predicted, heat decreased pollen vigour and pollen tube growth, reducing the number of pollen tubes that reached the base of the style. Unexpectedly, the effect of heat was not simply additive to that of pollen origin. Instead, the impact of pollen origin on pollen quality was only seen in control conditions. Under extreme heat, the quality effects of cross versus self-grains on pollen tube survival were indistinguishable, but much lower than under control conditions. Flowers that developed during extreme heat also produced less pollen than those that developed under control temperatures. Although we did not measure pollen dispersal via pollinators directly in this study, these reductions are expected to increase the risk of pollen quantity and quality limitation, as supported by the strong negative effects of heat on seed set.

### Heat effects on pollen quality limitation

Extreme heat has been found to decrease pollen vigour across multiple taxa (Aloni *et al.* 2001; Young *et al.* 2004; Jiang *et al.* 2019). In our hand pollination treatments, heat’s effect on pollen tube growth was evident as significant differences between heatwave and control treatments in the model-estimated rise (*r*) between pollen deposition and pollen tube survival. The rate of pollen tube survival in self-pollinated control flowers was approximately 27% lower than cross-pollinated flowers, showing a large, negative effect of pollen origin on pollen tube survival (see Supporting Information—**Table S1**). However, pollen origin effects were dwarfed and masked by heat, which decreased the rate of pollen tube survival in cross and self-pollinated flowers by approximately 71% and 77%, respectively (**see Supporting Information—Table S1**). While the difference was not statistically significant in heat-treated flowers, the pattern still reflects our prediction that the heatwave-induced reduction in pollen vigour and pollen tube growth would contribute to qualitative pollen limitation.

Pollen origin has well-documented effects on pollen quality (Dogterom, Winston and Mukai 2000; Ramsey and Vaughton 2000; Aizen and Harder 2007; Harder and Aizen 2010; Harder, Aizen, Richards 2016), but in our study, somewhat surprisingly, heat nullified the benefits of cross-pollination for pollen tube survival in comparison to self-pollination. We highlight two possible explanations. One limitation of our study is that we could not parse the effects of cross and self-pollination during heat treatments without also affecting the pistil. We may have seen similar responses of cross and self-pollination in heat-treated flowers because heat can act synergistically in *Brassica napus* on pollen and pistils to prevent micropyle guidance which may have affected cross and self-pollen equally (tested only with cross pollen: Young *et al.* 2004). Alternatively, environmental stress in pollen donors can slow pollen tube growth in recipient plants (Mutikainen and Delph 1996; Delph, Johannsson and Stephenson 1997; Aizen and Raffeale 1998). As heat affected both cross and self-pollen, it may have slowed pollen tube growth so that differences were still indistinguishable after 24 h.

Regardless of the mechanism, the equivalent performance of cross and self-grains implies that pollen origin may be less relevant to seed production during heatwaves in self-compatible plant species. Indeed, for total seed production and fertilization, pollen dispersal within (self-pollination) and among (cross-pollination) plants should be equally successful so long as pollen grains are still able to be dispersed (Hemberger *et al.* 2023). Our result contrasts with results for *Vicia faba* for which cross pollen sired more seeds following heat treatment than self-pollen (Bishop *et al.* 2017). Indeed *V. faba* plants are more dependent on cross-pollination during heatwaves (Bishop *et al*. 2016). However, this study also used a much more modest heatwave temperature (30 °C vs. our 35 °C) which may demonstrate a context dependency of the intensity of heat on pollination and differential mating success between cross and self-pollen.

### Heat effects on pollen quantity limitation

Numerous studies have demonstrated the negative impacts of modest heat on pollen production (e.g. 33°C–38 °C; Prasad *et al.* 1999, 2002; Hedhly *et al.* 2009; Hedhly 2011), which can contribute to quantitative pollen limitation. In our study, the number of pollen grains produced per flower decreased by 20% in plants exposed to 3 days of heat stress. With our hand pollination experiments, we made a concerted effort to create a full range of pollen deposition among flowers that would effectively characterize the relationship between the numbers of grains deposited and the numbers of pollen tubes reaching the ovary. Despite this effort, we were unable to achieve high pollen deposition in heat-treated plants to match that of control plants (Fig. 3), likely due to reduced pollen production from our heat treatment. We acknowledge that it could also be due to changes in stigma receptivity, pollen adherence to a stigmatic surface, or failed pollen germination (Hedhly *et al.* 2009), but this effect was not previously found from reciprocal crossing with experiments in *Brassica napus* (Young *et al.* 2004).

In plants that depend on animal vectors for pollination, reduced pollen production may also interact with heat’s effects on pollinators to further depress pollen dispersal and induce pollen quantity limitation. When less pollen is available in a pollen pool, stigmas of receptive flowers receive less pollen per visit even when pollinator visitation is not limited (Cresswell 1999). Heat may further reduce average pollen deposition by reduced pollen availability, which may also compound pollen limitation when heat reduces flower visitation rates and the number of foraging bouts of pollinators (Walters *et al.* 2022; Hemberger *et al.* 2023). There may be thermal conditions that increase pollinator activity and elevate flower visitation (Ma, Ma and Pincebourde 2021) so that individual flowers overcome pollen limitation, but other studies have indicated that plants exposed to heat produce fewer floral resources for visiting pollinators (Moss and Evans 2022; Hemberger *et al.* 2023; Walters *et al.* 2024). However, when heat exceeds pollinator thermal optima these benefits likely diminish (Walters *et al.* 2022; Hemberger *et al.* 2023). Additional research is crucially needed to measure heat’s relative effect on pollination via its impact on pollinator foraging, and its impact on flowers as it relates to pollen limitation.

### Limitations of *Brassica napus*

Our research evaluated a single plant species, *Brassica napus*, with a self-compatible mixed mating system, but species with different mating systems may be differentially affected by decreased pollen production and pollen vigour caused by heat. For example, autogamously pollinated species (those that obligately self) may be even more vulnerable because their flowers produce substantially less pollen compared to plant species that depend on pollen vectors (Harder and Johnson 2023), and this reduction cannot be offset by the receipt of cross pollen. In self-incompatible plant species, it seems logical that heat will exacerbate pollen limitation based on our observed changes in pollen production and that cross-pollination in our experiments yielded a substantially lower pollen tube survival rate and seed set compared to plants in the control treatments. Other plants with mixed mating systems show an increase in cross-pollen siring success relative to self-pollen in response to heat when pollen competed simultaneously (Bishop *et al.* 2017). We did not evaluate the rate of pollen tube survival from the perspective of competing cross and self-pollen. Comparing cross and self-pollination mixes to understand whether this pattern holds true when cross and self-pollen compete for ovule fertilization simultaneously would be important to expand our framework. Furthermore, it may explain expectations of pollen limitation for flowering plants with more complex mating systems and complex responses to heat (e.g. cryptic self-incompatibility: Cruzan and Barrett 2016; heat-induced self-incompatibility: Distefano *et al.* 2009; disrupted self-incompatibility response: Distefano *et al.* 2018).

## Conclusion

Our work demonstrated how extreme heat decreases pollen availability and vigour, which compounds to exacerbate both pollen quantity and quality limitation to limit reproductive success in *Brassica napus*. Although *B. napus* is highly sensitive to extreme temperatures, overwhelming evidence among flowering plant taxa indicates deleterious effects of heat stress on floral reproduction during the flowering stage (Hedhly *et al.* 2009; Zinn *et al.* 2010; Bishop *et al.* 2017). Our results and expansions of the pollen limitation framework are prudent, as they inform expectations of how the plant mating environment may change (from the plant’s perspective) during heatwaves. Indeed, recent intense heatwaves have overlapped with the flowering periods of important agricultural crops in different world regions with devastating effects for yield (Bal *et al.* 2022; White *et al.* 2023). The role of heat-induced pollen limitation was not specifically explored in these cases, but our results suggest that heat-induced pollen limitation may be a contributing factor.

Importantly, our finding that heat nullifies pollen origin differences in pollen tube survival is pertinent because cross-pollination success and mating are the medium by which plant populations can adapt to a changing environment (Eckert *et al.* 2010; Harder and Aizen 2010). Because heat seemingly disrupts the differential effects of pollen origin on pollen tube survival, heat may: (i) limit the number of individuals in a population who are able to mate; (ii) increase the incidence of self-pollination and inbreeding depression; and (iii) prevent adaptation to additional selection pressures via disrupted mating. Future research should aim to quantify how heat during flowering impacts demographic processes and affects selection by additional selection pressures.

As the frequency, intensity, and duration of heatwaves increase presently and into the future (Meehl and Tebaldi 2004; Stillman 2019; Thiery *et al*. 2021), plants that flower during periods of extreme heat may not receive sufficient pollination to overcome heat’s negative quantitative and qualitative effects on flowers and thus experience pollen limitation. Such heat-induced pollen quantity and quality limitation will undoubtedly be an important force shaping the mating of flowering plant species in a rapidly warming world.

## Supporting Information

The following additional information is available in the online version of this article – Rosenberger_et_al_aobp_supporting_information.pdf

plae045_suppl_Supplementary_Materials

## Data Availability

The data and R code used for analyses in this study is publicly available the Dryad Digital Repository and can be found at https://doi.org/10.5061/dryad.tmpg4f572

## Appendix 1 – Justification of asymptote selection

We used our analysis of pollen tube survival in a non-linear model with independent rise and asymptote parameters (see Methods). We used this same model structure (Equation 1) a priori to parameterize the optimal asymptote which is the maximum number of pollen tubes a flower can support. The estimates from this model are presented in the table below.

**Table:**

Heat treatment	Flower treatment	Mean asymptote estimate	Standard error
Control	Cross	25.8	2.31
Control	Self	23.8	2.16
Heat	Cross	12.2	4.54
Heat	Self	14.8	5.71

## References

[CIT0001] Aizen MA , Harder LD. 2007. Expanding the limits of the pollen-limitation concept: effects of pollen quantity and quality. Ecology 88:271–281. 17479745 10.1890/06-1017

[CIT0002] Aizen MA , Raffaele E. 1998. Flowering-shoot defoliation affects pollen grain size and postpollination pollen performance in *Alstroemeria aurea*. Ecology 79:2133–2142.

[CIT0003] Aloni B , Peet M, Pharr M, Karni L. 2001. The effect of high temperature and high atmospheric CO_2_ on carbohydrate changes in bell pepper (*Capsicum annuum*) pollen in relation to its germination. Physiologia Plantarum 112:505–512. 11473710 10.1034/j.1399-3054.2001.1120407.x

[CIT0004] Ashman T-L , Knight TM, Steets JA, Amarasekare P, Burd M, Campbell DR, Dudash MR, Johnston MO, Mazer SJ, Mitchell RJ, et al. 2004. Pollen limitation of plant reproduction: Ecological and evolutionary causes and consequences. Ecology 85:2408–2421.

[CIT0005] Bal SK , Prasad JVNS, Singh VK. 2022. Heat wave 2022 Causes, impacts and way forward for Indian Agriculture. Hyderabad: Technical Bulletin No. ICAR/CRIDA/ TB/01/2022, ICAR-Central Research Institute for Dryland Agriculture.

[CIT0006] Barrett SCH. 2002. Sexual interference of the floral kind. Heredity 88:154–159. 11932774 10.1038/sj.hdy.6800020

[CIT0008] Battisti DS , Naylor RL. 2009. Historical warnings of future food insecurity with unprecedented seasonal heat. Science 323:240–244. 19131626 10.1126/science.1164363

[CIT0009] Bishop J , Jones HE, Lukac M, Potts SG. 2016. Insect pollination reduces yield loss following heat stress in faba bean (*Vicia faba* L.). Agriculture, Ecosystems & Environment 220:89–96. 10.1016/j.agee.2015.12.007 PMC4767028 26989276

[CIT0010] Bishop J , Jones HE, O’Sullivan DM, Potts SG. 2017. Elevated temperature drives a shift from selfing to outcrossing in the insect-pollinated legume, faba bean (*Vicia faba*). Journal of Experimental Botany 68:2055–2063. 27927999 10.1093/jxb/erw430 PMC5429019

[CIT0011] Brooks ME , Kristensen K, van Benthem KJ, Magnusson A, Berg CW, Nielsen A, Skaug HJ, Maechler M, Bolker BM. 2017. glmmTMB Balances Speed and Flexibility Among Packages for Zero-inflated Generalized Linear Mixed Modeling. *The R Journal* 9: 378–400.

[CIT0012] Cresswell JE. 1999. The influence of nectar and pollen availability on pollen transfer by individual flowers of oil-seed rape (*Brassica napus*) when pollinated by bumblebees (*Bombus lapidarius*). Journal of Ecology 87:670–677.

[CIT0013] Cruzan MB. 1986. Pollen tube distributions in *Nicotiana glauca*: evidence for density dependent growth. American Journal of Botany 73:902–907.

[CIT0014] Cruzan MB. 1989. Pollen tube attrition in *Erythronium grandiflorum*. American Journal of Botany 76:562–570.

[CIT0015] Cruzan MB , Barrett SCH. 2016. Postpollination discrimination between self and outcross pollen covaries with the mating system of a self-compatible flowering plant. American Journal of Botany 103:568–576. 26507113 10.3732/ajb.1500139

[CIT0016] Delph LF , Johannsson MH, Stephenson AG. 1997. How environmental factors affect pollen performance: Ecological and evolutionary perspectives. Ecology 78:1632–1639.

[CIT0017] Distefano G , Caruso M, La Malfa S, Gentile A, Tribulato E. 2009. Histological and molecular analysis of pollen–pistil interaction in clementine. Plant Cell Reports 28:1439–1451. 19636563 10.1007/s00299-009-0744-9

[CIT0018] Distefano G , Gentile A, Hedhly A, La Malfa S. 2018. Temperatures during flower bud development affect pollen germination, self-incompatibility reaction and early fruit development of clementine (*Citrus clementina* Hort. ex Tan.). Plant Biology (Stuttgart, Germany) 20:191–198. 29106780 10.1111/plb.12656

[CIT0019] Dogterom MH , Winston ML, Mukai A. 2000. Effect of pollen load size and source (self, outcross) on seed and fruit production in highbush blueberry cv. ‘Bluecrop’ (*Vaccinium corymbosum*; Ericaceae). American Journal of Botany 87:1584–1591. 11080108

[CIT0020] Eckert CG , Kalisz S, Geber MA, Sargent R, Elle E, Cheptou P-O, Goodwillie C, Johnston MO, Kelly JK, Moeller DA, et al. 2010. Plant mating systems in a changing world. Trends in Ecology & Evolution 25:35–43. 19683360 10.1016/j.tree.2009.06.013

[CIT0021] Harder LD , Aizen MA. 2010. Floral adaptation and diversification under pollen limitation. Philosophical Transactions of the Royal Society of London, Series B: Biological Sciences 365:529–543. 20047878 10.1098/rstb.2009.0226 PMC2838256

[CIT0022] Harder LD , Aizen MA, Richards SA. 2016. The population ecology of male gametophytes: The link between pollination and seed production (JM Gomez, Ed.). Ecology Letters 19:497–509. 26970246 10.1111/ele.12596

[CIT0023] Harder LD , Aizen MA, Richards SA, Joseph MA, Busch JW. 2016. Diverse ecological relations of male gametophyte populations in stylar environments. American Journal of Botany 103:484–497. 26933012 10.3732/ajb.1500269

[CIT0024] Harder LD , Johnson SD. 2023. Beyond pollen:ovule ratios: evolutionary consequences of pollinator dependence and pollination efficiency for pollen and ovule production in angiosperms. American Journal of Botany 110:e16177. 37146240 10.1002/ajb2.16177

[CIT0027] Harvey JA , Tougeron K, Gols R, Heinen R, Abarca M, Abram PK, Basset Y, Berg M, Boggs C, Brodeur J, et al. 2023. Scientists’ warning on climate change and insects. Ecological Monographs 93:e1553.

[CIT0029] Hedhly A. 2011. Sensitivity of flowering plant gametophytes to temperature fluctuations. Environmental and Experimental Botany 74:9–16.

[CIT0030] Hedhly A , Hormaza JI, Herrero M. 2005. The effect of temperature on pollen germination, pollen tube growth, and stigmatic receptivity in peach. Plant Biology (Stuttgart, Germany) 7:476–483. 16163612 10.1055/s-2005-865850

[CIT0031] Hedhly A , Hormaza JI, Herrero M. 2009. Global warming and sexual plant reproduction. Trends in Plant Science 14:30–36. 19062328 10.1016/j.tplants.2008.11.001

[CIT0032] Hemberger JA , Rosenberger NM, Williams NM. 2023. Experimental heatwaves disrupt bumblebee foraging through direct heat effects and reduced nectar production. Functional Ecology 37:591–601.

[CIT0033] Jamieson MA , Trowbridge AM, Raffa KF, Lindroth RL. 2012. Consequences of climate warming and altered precipitation patterns for plant-insect and multitrophic interactions. Plant Physiology 160:1719–1727. 23043082 10.1104/pp.112.206524 PMC3510105

[CIT0034] Jiang Y , Lahlali R, Karunakaran C, Warkentin TD, Davis AR, Bueckert RA. 2019. Pollen, ovules, and pollination in pea: Success, failure, and resilience in heat. Plant, Cell & Environment 42:354–372. 10.1111/pce.13427 30136298

[CIT0035] Jiménez MA , Jaksic FM, Armesto JJ, Gaxiola A, Meserve PL, Kelt DA, Gutiérrez JR. 2011. Extreme climatic events change the dynamics and invasibility of semi-arid annual plant communities. Ecology Letters 14:1227–1235. 21988736 10.1111/j.1461-0248.2011.01693.x

[CIT0036] Jóhannsson MH , Gates MJ, Stephenson AG. 1998. Inbreeding depression affects pollen performance in Cucurbita texana. Journal of Evolutionary Biology 11:579–588.

[CIT0037] Kao TH , McCubbin AG. 1996. How flowering plants discriminate between self and non-self pollen to prevent inbreeding. Proceedings of the National Academy of Sciences of the United States of America 93:12059–12065. 8901531 10.1073/pnas.93.22.12059 PMC37941

[CIT0038] Knight TM , Steets JA, Vamosi JC, Mazer SJ, Burd M, Campbell DR, Dudash MR, Johnston MO, Mitchell RJ, Ashman T-L. 2005. Pollen limitation of plant reproduction: Pattern and process. Annual Review of Ecology, Evolution, and Systematics 36:467–497.

[CIT0039] Koti S , Reddy KR, Reddy VR, Kakani VG, Zhao D. 2005. Interactive effects of carbon dioxide, temperature, and ultraviolet-B radiation on soybean (*Glycine max* L.) flower and pollen morphology, pollen production, germination, and tube lengths. Journal of Experimental Botany 56:725–736. 15611147 10.1093/jxb/eri044

[CIT0040] Lancashire PD , Bleiholder H, Van den Boom T, Langelueddeke P, Stauss R, Weber E, Witzenberger. 1991. A uniform decimal code for growth stages of crops and weeds. Annals of Applied Biology 119:561–601.

[CIT0041] Lenth RV , Bolker B, Buerkner P, et al. 2023. emmeans: Estimated marginal means, aka least-squares means.

[CIT0042] Lohani N , Singh MB, Bhalla PL. 2022. Short-term heat stress during flowering results in a decline in Canola seed productivity. Journal of Agronomy and Crop Science 208:486–496.

[CIT0043] Ma C , Ma G, Pincebourde S. 2021. Survive a warming climate: insect responses to extreme high temperatures. Annual Review of Entomology 66:163–184. 10.1146/annurev-ento-041520-074454 32870704

[CIT0044] Martin FW. 1959. Staining and observing pollen tubes in the style by means of fluorescence. Stain Technology 34:125–128. 13659240 10.3109/10520295909114663

[CIT0045] Meehl GA , Tebaldi C. 2004. More intense, more frequent, and longer lasting heat waves in the 21st Century. Science 305:994–997. 15310900 10.1126/science.1098704

[CIT0046] Monterroso VA , Wien HC. 1990. Flower and pod abscission due to heat stress in beans. Journal of the American Society for Horticultural Science 115:631–634.

[CIT0047] Moss E , Evans DM. 2022. Experimental climate warming reduces floral resources and alters insect visitation and wildflower seed set in a cereal agro-ecosystem. Frontiers in Plant Science 13:826205. 35283885 10.3389/fpls.2022.826205 PMC8905351

[CIT0048] Mutikainen P , Delph LF. 1996. Effects of herbivory on male reproductive success in plants. Oikos 75:353–358.

[CIT0049] Nicholson CC , Egan PA. 2020. Natural hazard threats to pollinators and pollination. Global Change Biology 26:380–391. 31621147 10.1111/gcb.14840

[CIT0050] Okamoto S , Odashima M, Fujimoto R, Sato Y, Kitashiba H, Nishio T. 2007. Self-compatibility in *Brassica napus* is caused by independent mutations in S-locus genes. The Plant Journal: for Cell and Molecular Biology 50:391–400. 17425715 10.1111/j.1365-313X.2007.03058.x

[CIT0051] Peet MM , Sato S, Gardner RG. 2002. Comparing heat stress effects on male-fertile and male-sterile tomatoes. Plant, Cell & Environment 21:225–231.

[CIT0052] Prasad PVV , Craufurd PQ, Summerfield RJ. 1999. Fruit number in relation to pollen production and viability in groundnut exposed to short episodes of heat stress. Annals of Botany 84:381–386.

[CIT0053] Prasad PVV , Boote KJ, Allen Jr LH, Thomas JMG. 2002. Effects of elevated temperature and carbon dioxide on seed-set and yield of kidney bean (*Phaseolus vulgaris* L.). Global Change Biology 8:710–721.

[CIT0054] Pincebourde S , Casas J. 2019. Narrow safety margin in the phyllosphere during thermal extremes. Proceedings of the National Academy of Sciences of the United States of America 116:5588–5596. 30782803 10.1073/pnas.1815828116 PMC6431205

[CIT0055] Pinheiro J , Bates D, R Core Team. 2023. nlme: Linear and nonlinear mixed effects models. R package v. 3.1-163

[CIT0056] Ramsey M , Vaughton G. 2000. Pollen quality limits seed set in *Burchardia umbellata* (Colchicaceae). American Journal of Botany 87:845–852. 10860915

[CIT0058] Shi W , Li X, Schmidt RC, Struik PC, Yin X, Jagadish SVK. 2018. Pollen germination and in vivo fertilization in response to high-temperature during flowering in hybrid and inbred rice. Plant, Cell & Environment 41:1287–1297. 10.1111/pce.13146 29336039

[CIT0059] Snow AA , Spira TP, Liu H. 2000. Effects of sequential pollination on the success of ‘fast’ and ‘slow’ pollen donors in *Hibiscus moscheutos* (Malvaceae). American Journal of Botany 87:1656–1659. 11080116

[CIT0060] Souto CP , Aizen MA, Premoli AC. 2002. Effects of crossing distance and genetic relatedness on pollen performance in *Alstroemeria aurea* (Alstroemeriaceae). American Journal of Botany 89:427–432. 21665638 10.3732/ajb.89.3.427

[CIT0061] Stillman JH. 2019. Heat waves, the new normal: summertime temperature extremes will impact animals, ecosystems, and human communities. Physiology (Bethesda, Md.) 34:86–100. 30724132 10.1152/physiol.00040.2018

[CIT0062] Thiery W , Lange S, Rogelj J, Schleussner C-F, Gudmundsson L, Seneviratne SI, Andrijevic M, Frieler K, Emanuel K, Geiger T, et al. 2021. Intergenerational inequities in exposure to climate extremes. Science 374:158–160. 34565177 10.1126/science.abi7339

[CIT0063] Walters J , Zavalnitskaya J, Isaacs R, Szendrei Z. 2022. Heat of the moment: extreme heat poses a risk to bee–plant interactions and crop yields. Current Opinion in Insect Science 52:100927. 35500861 10.1016/j.cois.2022.100927

[CIT0064] Walters J , Barlass MK, Fisher R, Isaacs R. 2024. Extreme heat exposure of host plants indirectly reduces solitary bee fecundity and survival. Proceedings Biological Sciences 291:20240714. 38889783 10.1098/rspb.2024.0714 PMC11285533

[CIT0065] White RH , Anderson S, Booth JF, Braich G, Draeger C, Fei C, Harley CDG, Henderson SB, Jakob M, Lau C-A, et al. 2023. The unprecedented Pacific Northwest heatwave of June 2021. Nature Communications 14:727. 10.1038/s41467-023-36289-3 PMC9910268 36759624

[CIT0066] Young LW , Wilen RW, Bonham‐Smith PC. 2004. High temperature stress of *Brassica napus* during flowering reduces micro‐ and megagametophyte fertility, induces fruit abortion, and disrupts seed production. Journal of Experimental Botany 55:485–495. 14739270 10.1093/jxb/erh038

[CIT0067] Zinn KE , Tunc-Ozdemir M, Harper JF. 2010. Temperature stress and plant sexual reproduction: uncovering the weakest links. Journal of Experimental Botany 61:1959–1968. 20351019 10.1093/jxb/erq053 PMC2917059

