# Supplementary material for: Heatwaves exacerbate pollen limitation through reductions in pollen production and pollen vigour
Source: AoB Plants. 2024 Sep 12;16(5):plae045. doi: 10.1093/aobpla/plae045 (PMC11447236; doi:10.1093/aobpla/plae045)
Supplement: plae045_suppl_Supplementary_Materials [file plae045_suppl_supplementary_materials.pdf]

Supplementary Materials

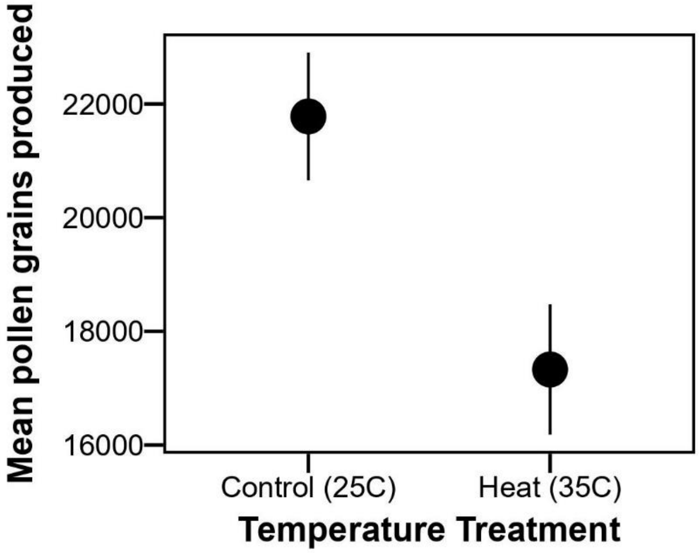

Figure S1 – Marginal mean number of pollen grains produced by a flower in response to temperature treatment ± SE.

Model	Link function	Parameter	Estimate ± SE	Z value	P value
Pollen tube survival	None	*r* - Heat / Self	$1.37x10^{-2} \pm 5.70x10^{-3}$		
		r - Control / Self	$4.43x10^{-2} \pm 5.94x10^{-3}$		
		r - Heat / Cross	$1.73x10^{-2} \pm 5.74x10^{-3}$		
		r - Control / Cross	$6.05x10^{-2} \pm 6.86x10^{-3}$		
Pollen production	None	Intercept	17328±1146	15.12	P<0.01
		Plant treatment	4452±1605	2.77	P<0.01
Seed set	log	Intercept	0.62±0.24	2.55	P<0.05
		Temperature treatment	2.13±0.31	6.84	P<0.01
		Pollen origin treatment	0.17±0.22	0.76	0.45
		Temperature - Pollen Origin Interaction	-0.19±0.26	-0.71	0.48

Table S1 - Parameter estimates from pollen tube models, pollen production, and seed set. The pollen tube model does not include significance values as tests from these analyses are reported in Tables 1 and 2.
